# Enhancement of cell-specific transgene expression from a Tet-Off regulatory system using a transcriptional amplification strategy in the rat brain

**DOI:** 10.1002/jgm.1178

**Published:** 2008-05

**Authors:** Beihui Liu, Shu Wang, Michael Brenner, Julian FR Paton, Sergey Kasparov

**Affiliations:** 1 Department of Physiology and Pharmacology, Bristol Heart Institute, School of Medical Sciences, University of BristolBristol BS8 1TD, UK; 2 Department of Biological Sciences, National University of SingaporeSingapore; 3 Institute of Bioengineering and Nanotechnology31 Biopolis Way, Singapore 138669, Singapore; 4 Department of Neurobiology, University of Alabama at BirminghamAL 35294-0021, USA

**Keywords:** gene transfer, tetracycline-regulated gene expression, CNS, lentiviral vectors, doxycycline, cell targeting, transcriptional amplification, neurons, astrocytes

## Abstract

**Background:**

The Tet-Off system uses a tetracycline-controlled transactivator protein (tTA) and a tetracycline-responsive promoter element (TRE) to regulate expression of a target gene. This system can be used to achieve regulatable transgene expression in specific cell types by employing a cell-specific promoter to drive tTA expression. Wide applications of this attractive approach are, however, hindered by relatively weak transcriptional activity of most cell-specific promoters. We report here the feasibility of using a transcriptional amplification strategy to overcome the problem.

**Methods and results:**

In the developed cell-type-specific, Tet-inducible lentiviral system, two distinct cellular promoters were tested, a human synapsin-1 promoter for neurons and a compact glial fibrillary acidic protein promoter for astroglial cells. Lentiviral vectors were constructed that contained two copies of one or the other of these two promoters. One copy was used to drive the expression of a chimeric transactivator consisting of a part of the transcriptional activation domain of the NF-κB p65 protein fused to the DNA-binding domain of the yeast GAL4 protein. The second copy of the cell-specific promoter was modified by introduction of the GAL4 binding sequences at its 5′ end. This copy was used to drive expression of tTA. A gene encoding a red fluorescent protein was cloned into another lentiviral vector under transcriptional control of TRE. Co-transduction with the two types of viral vectors provided doxycycline-regulated transgene expression in a neuron- or astrocyte-specific manner. Compared to control viruses without transcriptional amplification, our enhanced systems were approximately 8-fold more potent in cultured neurons and astroglial cells and at least 8- to 12-fold more potent in the rat brain *in vivo*.

**Conclusions:**

Our results demonstrate the effectiveness of the transcriptional amplification strategy in developing viral gene delivery systems that combine the advantages of specific cell type targeting and Tet-inducible expression. Copyright © 2008 John Wiley & Sons, Ltd.

## Introduction

The central nervous system (CNS) is a particularly complex organ, containing not only multiple types of neurons with different and sometimes opposing functions, but also oligodendrocytes, microglia and astrocytes. Astrocytes in particular have been of increasing interest as the extent of their roles in CNS function becomes understood 1,2. The existence of various types of cells in the CNS underscores the importance of restricting transgene expression to specified target cell types in the brain and has stimulated extensive studies to develop gene expression vectors with specific cellular promoters 3–5. In addition to directing specific gene expression, cell-type-specific promoters may be less likely to activate host cell defence machinery so that improved stability of gene expression can be expected 6,7. However, the main limitation of brain-cell-specific promoters is their relatively weak transcriptional activity compared with viral promoters 8–10.

An ideal gene delivery vector system for the CNS should also have the ability to efficiently control the timing of gene expression and the amount of expressed gene products. Among the different regulatable transgene expression systems that have been developed, the tetracycline-based regulatable system (Tet-system) 11 has been the most popular tool for controlling gene expression. There are two basic variants of the Tet-system; one utilises the tTA transactivator (‘Tet-Off’ system) and the other the rtTA transactivator (‘Tet-On’ system) 12,13. In the Tet-off system, tTA binds to the Tet-regulatable element (TRE) in the absence of doxycycline (Dox) and initiates transcription of the target gene from a promoter which incorporates the minimal cytomegalovirus (CMV) core promoter. The original TRE-based vectors suffered from disadvantages such as high basal ‘leak’ expression and low efficiency of regulation 14. With continuous attempts to overcome these problems, a modified TRE marketed by Clontech as ‘TRE-tight’ has been developed to achieve high inducibility combined with undetectable levels of leak expression 15. This TRE-tight promoter contains a 60-bp shortened CMV minimal promoter together with seven 19-bp tet operator sequences positioned in an optimised manner upstream of the TATA box 16. However, application of this TRE-tight system to cell-specific expression proved to be difficult: when used in combination with either the PRSx8 promoter 5 or the GAD67 promoter 17, we failed to achieve a sufficient level of gene expression (S. Kasparov and A. G.Teschemacher, unpublished observation; 18,19). The most logical explanation of this low activity was that a high level of tTA expression required for such a control cannot be supplied by the standard brain-cell-specific promoters.

A dual vector adenoviral Tet-system for tetracycline-controllable expression of transgenes in the brain was described by Harding *et al.* 20. The design had two main features: on one hand, it was possible to control gene expression in the brain by administering Dox into the drinking water. On the other, physically separating the transactivator elements from the TRE increased the control of gene expression. In addition, this design theoretically allows targeting of the transgene to a specific cellular phenotype when the Tet transactivator tTA or rtTA is placed under control of a cell-specific promoter. Later studies reported cell-specific Tet-off regulatable dual adenoviral systems where tTA was expressed under the control of neuronal- or glial-specific promoters 18,21. However, in both instances unpotentiated cell-type-specific promoters and the original TRE, rather than TRE-tight, were employed. This led to low levels of tTA expression and leak expression with appearance of the transgenes in unsolicited cell types 4.

In order to overcome these limitations, we have employed a relatively generalisable method, a two-step transcriptional amplification (TA) strategy. The method utilises artificial transcriptional activators to enhance transgene expression from potentially any cell-type-specific promoter 8,22,23. This strategy involves cell-type-specific expression of a strong chimeric activator GAL4p65 consisting of the transactivation domain of nuclear factor-kappaB (NFκB) p65 protein and the DNA-binding domain of the GAL4 protein from yeast. GAL4p65 then binds to multiple Gal4-binding sites placed upstream of the second copy of the same cell-specific promoter, leading to a massively amplified expression of the transgene. We hypothesised that tTA expressed using this approach would efficiently control expression of TRE-tight-based cassettes. As a proof of principle, we have tested the two-step TA method using two relatively weak cell-type-specific promoters. One is the 495-bp SYN promoter, which has been extensively characterised and shown to drive neuron-specific expression in various regions in the brain 10,24. The other is a human glial fibrillary acidic protein (GFAP) that drives gene expression in astrocytes 25. To accommodate the limited cloning capacity of lentiviruses, we used a compact version of the standard 2.2-kb GFAP promoter made by deleting 5′ nucleotides − 2163 to − 758 and an internal segment from − 1255 to − 133. The resultant 681-bp promoter, GfaABC_1_D, has expression properties in transgenic mice indistinguishable from the 2.2-kb version (unpublished observations).

## Materials and methods

### Construction of lentiviral plasmids

Five lentiviral plasmids were constructed based on the improved lentiviral shuttle vector pTYF-SW-Linker backbone 26. To generate the LV-Tretight-DsRed2 shuttle vector, the Tretight-DsRed2 fragment containing the modified Tet-responsive promoter and the red fluorescent protein (DsRed2) gene was excised from pTRE-Tight-DsRed2 (Clontech) with *Xho*I*/Not*I and cloned into pTYF-SW-Linker. The LV-1 × SYN-tTA shuttle vector was obtained by replacing the EGFP fragment in pGBS-SYN-EGFP 22 with tTA. To construct the LV-2 × SYN-tTA shuttle vector, tTA was amplified by polymerase chain reaction (PCR) and ligated into *Spe*I/*Not*I sites of pTYF-2 × SYN-EGFP 22 after the EGFP fragment had been removed. The LV-1 × GfaABC_1_D-tTA shuttle vector was produced by replacing the SYN fragment in the LV-1 × SYN-tTA shuttle vector with the GfaABC_1_D PCR product between the *Mlu*I and *Bam*HI sites. Three cloning steps were necessary to generate the LV-2 × GfaABC_1_D-tTA shuttle vector. First, the SYN promoter in pSYN-GAL4p65 22 was replaced by the GfaABC_1_D PCR product between the *Ase*I and *Nhe*I sites. The plasmid was then digested with *Ase*I, filled in with Klenow enzyme, followed by *Mlu*I digestion. The fragment encoding the GfaABC_1_D promoter, GAL4p65 and SV40pA was then isolated and cloned into *Eco*RV/*Mlu*I-treated pTYF-SW linker. Finally, the *Nhe*I/blunt/*Xho*I fragment from the LV-1 × GfaABC_1_D-tTA shuttle vector was inserted into the resultant plasmid from the above two steps previously treated with *Mlu*I/blunt/*Not*I.

### Production of lentiviral (LV) vectors

The LV system used in this study is derived from HIV-1 and pseudotyped with the vesicular stomatitis virus coat glycoprotein. LV stocks were produced by transient co-transfection of the shuttle plasmids, the packaging vector pNHP, and the envelope plasmid pHEF-VSVG in HEK293FT cells. Viral concentration and titration were carried out as described earlier 27.

### Cell culture and *in vitro* LV vector transduction

The *in vitro* transduction experiments were carried out in a neurone-derived rat pheochromocytoma PC12 cell line and a 1321N1 glial cell line from human brain astrocytoma. PC12 cells were grown in Dulbecco's modified Eagle's medium (DMEM) supplemented with 10% heat-inactivated fetal bovine serum (FBS) and 5% horse serum. 1321N1 cells were cultured in DMEM supplemented with 10% heat-inactivated FBS. The cells were split and plated in 24-well plates at a cell density of 5 × 10^4^/well with 0.5 ml culture medium. After 24 h, cells were transduced overnight with appropriate lentiviruses in the presence of polybrene (8 µg/ml). Cells were then washed in phosphate-buffered saline (PBS) and were cultured in DMEM for a further 48 h. For each virus combination, three wells were transduced.

### Delivery of LV vectors into the rat hypoglossal motor nucleus *in vivo*

Male Wistar rats (250–300 g) were used. All procedures were carried out according to the Home Office Animals Scientific Procedures Act 1986, UK. Animals were deeply anaesthetised with an intramuscular injection of ketamine (60 mg/kg) and medetomidine (250 µg/kg). They were placed in a stereotaxic head holder and the caudal dorsal medulla was exposed through a midline incision in the dorsal neck. A total of six microinjections of viral vector were made bilaterally at the level of the calamus scriptorius and 400 µm rostral and caudal to it, 300–500 µm from the midline and 450–550 µm ventral to the dorsal surface of the medulla. The injection rate was 0.5 µl/min and the injection needle was allowed to remain *in situ* for 5 min before being slowly retracted at the end of each injection. The wound was sutured, cleaned and treated with antiseptic powder. Medetomidine anaesthesia was reversed with a subcutaneous injection of atipamezole (1 mg/kg). Animals were returned to individual cages for recovery, and kept with normal rat chow and drinking water *ad libitum* on a 12 h light/12 h dark cycle. Seven days after injection, rats were terminally anaesthetised (sodium pentobarbital, 100 mg/kg, intramuscularly) and perfused intracardially with 4% formaldehyde in 0.1 M PBS (pH 7.4). Brainstems were then removed, postfixed in the same fixative for 2–4 h before they were placed in 20% sucrose overnight. Serial 40 µm sections were cut on a freezing microtome and kept in 0.1 m PBS. For quantitative analysis, three rats from each group were used to examine the number of DsRed2-positive cells. Four sections surrounding the injection tract for each rat were selected randomly and three fields from each section were used for cell counting.

### Immunohistochemistry analysis

Free-floating formaldehyde-fixed sections (as described above) were washed for 20 min in 0.1 M PBS at pH 7.4 containing 0.2% Triton X-100. Sections were then incubated overnight with a monoclonal antibody against neuron-specific nuclear protein (NeuN) or glial fibrillary acidic protein (GFAP) (both from Chemicon International, USA; dilution 1 : 500) and 5% normal horse serum (NHS) in PBS. This was followed by 4 h incubations in biotinylated donkey-anti mouse F(ab)_2_ fragments (1 : 500, Jackson Immunolabs, PA, USA) and 2% NHS in PBS, then ExtrAvidin-FITC in PBS (1 : 1000, Sigma). Washes were performed between incubations (PBS, 3 × 5 min), at room temperature. Sections were then collected on gelatin-coated slides with Vectashield non-quenching mounting medium (Vector Labs, CA, USA). Confocal images were obtained using a Leica confocal SP microscope. Images were taken at 1–2 µm intervals throughout the thickness of the section. The two channels (DsRed2 and FITC) were scanned separately using 488 and 543 nm excitation laser lines to avoid ‘bleed’ of fluorescence between channels and merged using Leica software.

### Statistical analysis

Unpaired *t* test was applied for comparisons between two groups. The differences were considered significant at *p* < 0.05. All values in the text and figures refer to mean ± standard deviation (SD).

## Results

### Construction of lentiviral (LV) vectors

**Figure 1:**
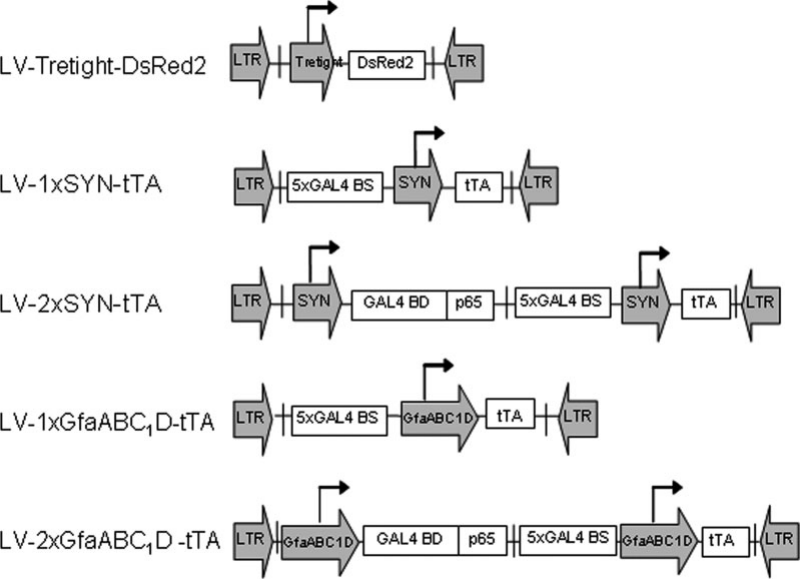
Schematic drawing of lentiviral vectors used in this study. LTR, lentiviral long terminal repeat; Tretight, a modified tetracycline-responsive promoter derived from pTRE-tight-DsRed2 (Clontech); DsRed2, red fluorescent protein; 5 × GAL4BS, five tandem GAL4 binding sites; SYN, human synapsin 1 promoter; GfaABC_1_D, a compact glial fibrillary acidic protein promoter; GAL4p65, a chimeric transactivator consisting of a part of the transactivatin domain of the murine NF-κB p65 protein fused to the DNA-binding domain of the GAL4 protein from yeast; tTA, tetracycline-controlled transactivator protein

Five self-inactivated HIV-derived LV vectors (Figure 1) were constructed for this study. (1) LV-Tretight-DsRed2, in which the DsRed2 reporter gene is under control of the TRE-tight promoter; (2) LV-1 × SYN-tTA, in which the Tet-Off system transactivator tTA gene is driven by the SYN promoter only; (3) LV-2 × SYN-tTA, a dual expression cassette containing SYN-driven tTA and the SYN-driven GAL4p65 in a single backbone; (4) LV-1 × GfaABC_1_D-tTA, in which the tTA gene is transcribed by the GfaABC_1_D promoter only; and (5) LV-2 × GfaABC_1_D-tTA, a dual expression cassette containing GfaABC_1_D-driven tTA and the GfaABC_1_D-driven GAL4p65 in a single backbone. Thus, the combinations of LV-Tretight-DsRed2 + LV-2 × SYN-tTA and LV-Tretight-DsRed2 + LV-2 × GfaABC_1_D-tTA constitute the TA-enhanced lentiviral neuronal- or glial-specific Tet-regulatable systems. LV-Tretight-DsRed2 + LV-1 × SYN-tTA and LV-1 × GfaABC_1_D-tTA, without TA, served as controls.

### *In vitro* analysis of gene expression

As a starting point, we tested the ‘tightness’ of the TRE-tight element using LV-Tretight-DsRed2 *in vitro*. In the absence of Dox, transgene expression from LV-Tretight-DsRed2 (multiplicity of infection (MOI) = 5) was undetectable in either neuronal PC12 cells or glial 1321N1 cells, confirming no leak expression of the vectors in the tested cells (data not shown).

**Figure 2:**
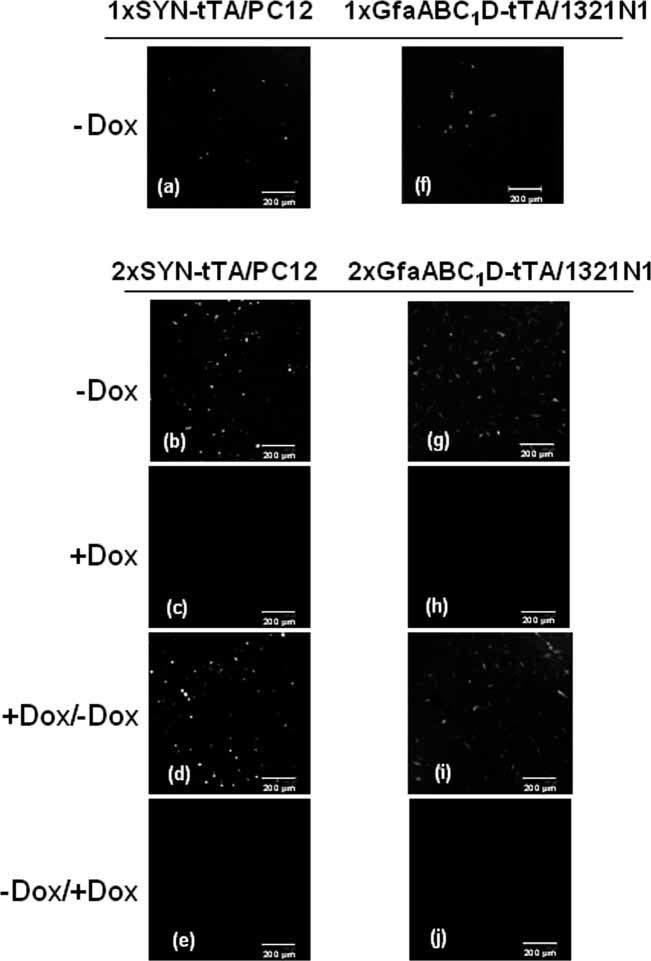
DsRed2 transgene expression *in vitro* in neuronal PC12 (a–e) and astroglial 1321N1 (f–j) cells. Cells were transduced with LV-Tretight-DsRed2 and either LV-1 × SYN-tTA (a), LV-2 × SYN-tTA (b, c, d, e), LV-1 × GfaABC1D-tTA (f), or LV-2 × GfaABC1D-tTA (g, h, i, j). The ratio between the trasactivator and Tre viruses was 1 : 1, with a total viral MOI of 5 per well. (a, b, f, g) Forty-eight hours after transduction without Dox treatment. DsRed2 expression was observed in all four groups. (c, h) Forty-eight hours after transduction in the presence of Dox. DsRed2 expression was completely repressed. (d, i) Dox was present for 48 h after transduction followed by a change to Dox-free medium and culturing for 4 more days. Repressed DsRed2 gene was retrieved maximally. (e, j) Dox was absent for 48 h after transduction followed by a change to Dox-containing medium and culturing for 4 more days. Note DsRed2 expression was completely abolished

**Figure 3:**
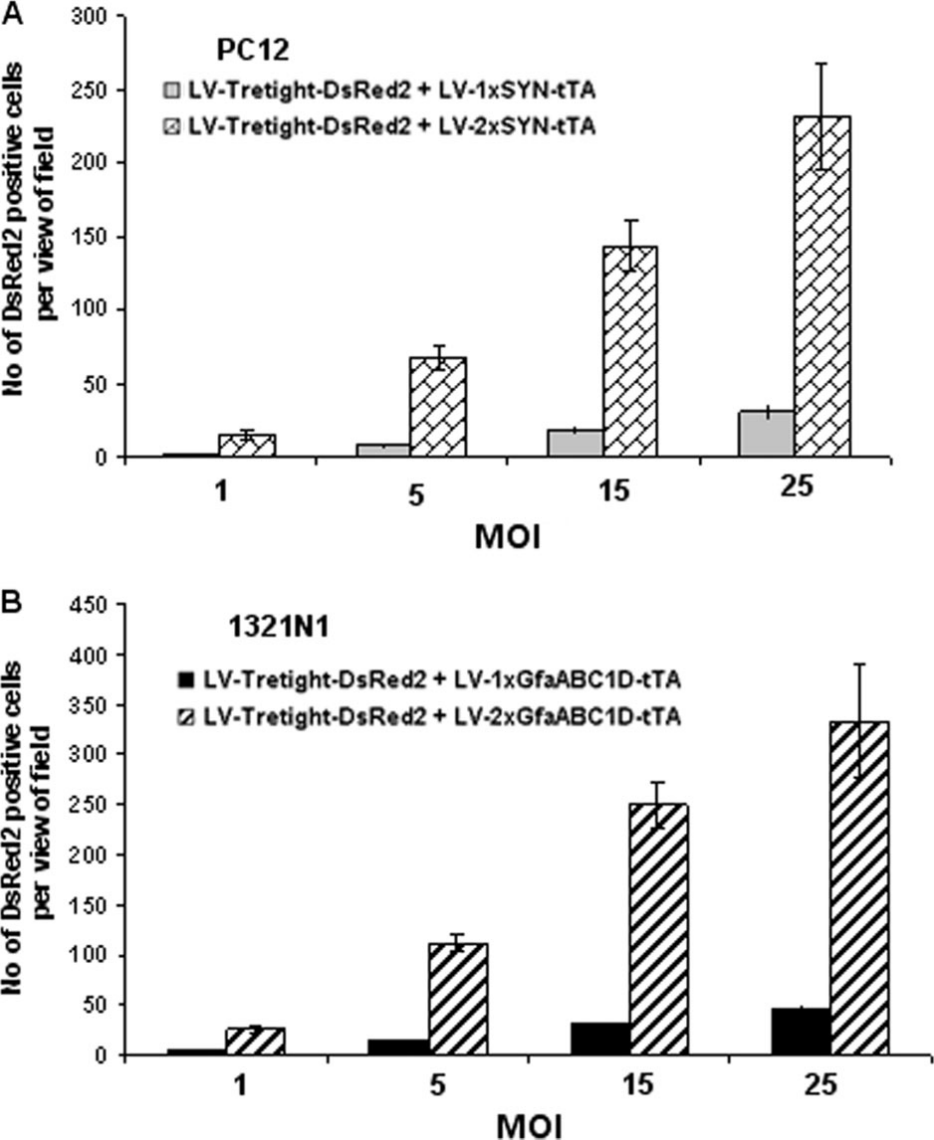
Quantification of *in vitro* transgene expression from transcriptional amplification-enhanced Tet-Off regulatory systems at MOIs of 1, 5, 15 and 25. (A) SYN-containing system in neuronal PC12 cells. (B) GfaABC1D-containing system in astroglial 1321N1 cells. Numbers of DsRed2-positive cells per field of view were counted under 100× magnification. Six fields were selected randomly for cell counting. The error bars indicate the standard deviations

PC12 cells were then co-transduced with LV-Tretight-DsRed2 and either LV-1 × SYN-tTA or LV-2 × SYN-tTA while 1321N1 cells were co-transduced with LV-Tretight-DsRed2 and either LV-1 × GfaABC_1_D-tTA or LV-2 × GfaABC_1_D-tTA. The ratio between the two co-transduced vectors was 1 : 1 and the total MOI for each pair of the viral vectors was 1, 5, 15 or 25. At all of the MOIs tested, TA-enhanced systems drove significantly increased DsRed2 transgene expression as compared to the non-enhanced systems (Figures 2 and 3), with expression from the SYN-driven TA-enhanced system being increased approximately 8-fold (at MOIs of 5, 15 and 25) (Figure 3A) and expression from the GfaABC_1_D-driven TA-enhanced system being increased approximately 7-fold at MOIs of 5, 15 and 25 (Figure 3B). Therefore, the extent of enhancement was similar across a wide range of MOIs. Expression of DsRed2 in PC12 and 1321N1 cells was completely inhibited in the continuous presence of Dox (2 µg/ml) (Figures 2c and 2h) or in the absence of Dox for 2 days followed by Dox treatment for 4 days (Figures 2e and 2j). The inhibition of gene expression resulting from Dox treatment for 2 days (Figures 2c and 2h) could be restored to the uninhibited levels by cell culturing in the absence of Dox for 4 days (cf. Figures 2b and 2g to 2d and 2j). These results indicate that TA enhancement potentiates tTA/TRE-tight-driven transgene expression without compromising its sensitivity to Dox. To investigate the cell-type specificity of the TA-enhanced system *in vitro*, we co-transduced PC12 and 1321N1 cells with LV-Tretight-DsRed2/LV-2 × GfaABC_1_D-tTA and LV-Tretight-DsRed2/LV-2 × SYN-tTA, respectively (molar ratio 1 : 1; MOI 5) in a separate experiment. No gene expression was observed from both cell lines (data not shown) indicating that the neuronal and glial characteristics of the SYN and the GfaABC_1_D promoter in the TA-enhanced system were well retained.

### *In vivo* analysis of gene expression

**Figure 4:**
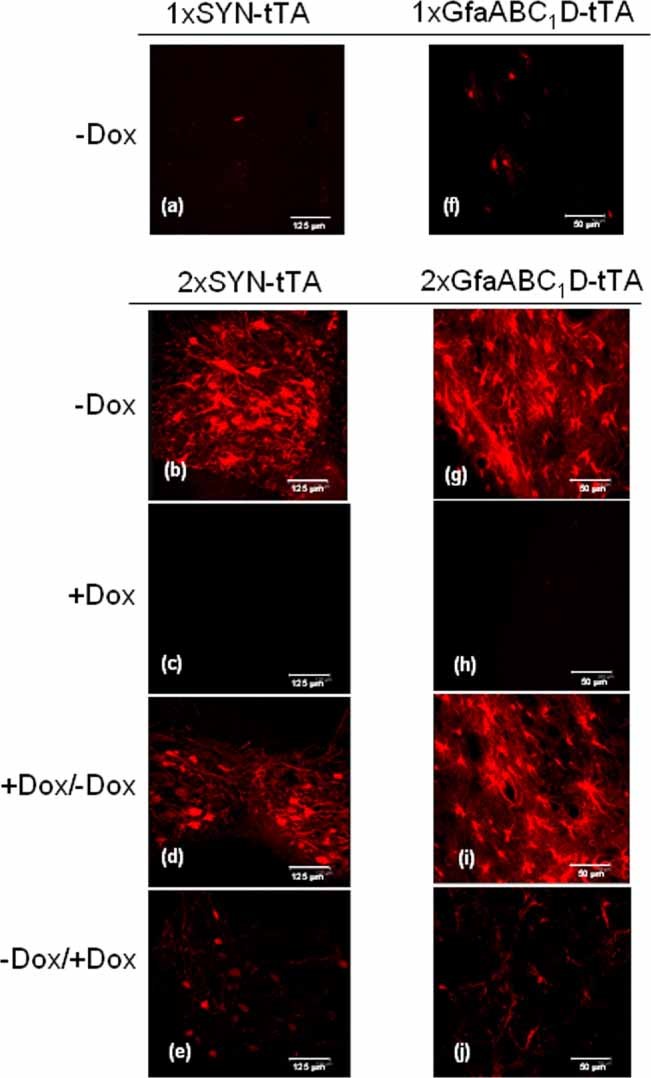
DsRed2 transgene expression in the hypoglossal motor nucleus of the rat brain. LV-Tretight-DsRed2 was injected with either LV-1 × SYN-tTA (a), LV-2 × SYN-tTA (b, c, d, e), LV-1 × GfaABC1D-tTA (f), or LV-2 × GfaABC1D-tTA (g, h, i, j). The ratio between the transactivator-expressing and Tre viruses was 4 : 1 and a total of 6 × 10^6^ IU of viruses was injected per rat. (a, b, f, g) Seven days after injection and without Dox treatment. DsRed2 expression was observed in all four groups. (c, h) Virus-injected rats drank Dox-containing water for 7 days. DsRed2 expression was completely repressed. (d, i) Dox treatment for the first 7 days after injection followed by Dox-free water for another 7 days. Repressed DsRed2 gene was retrieved maximally. (e, j) Dox-free water for the first 7 days after injection followed by Dox-containing water for another 7 days. Note DsRed2 expression was almost completely abolished

**Figure 5:**
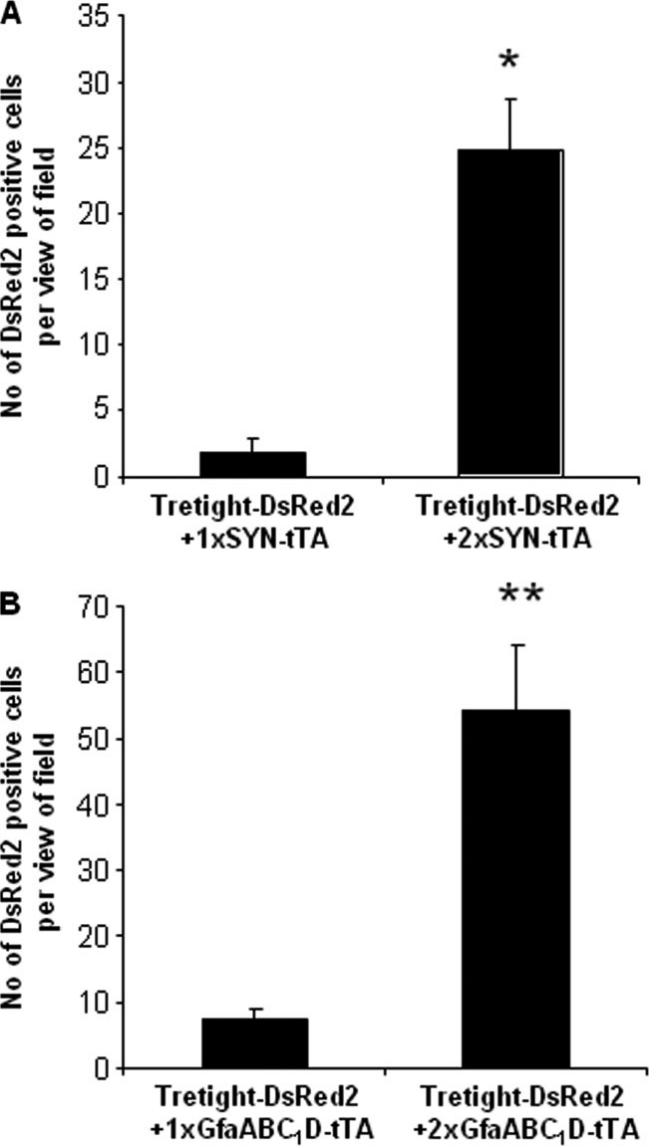
Quantification of *in vivo* transgene expression from TA-enhanced Tet-Off regulatory systems. Viruses were injected into the rat brain as described, see legend for Figure 4. (A) SYN-based system. (B) GfaABC_1_D-based system. Seven days after injection, numbers of DsRed2-positive cells per view of field were counted under 400× magnification. For each rat, four 40 µm coronal sections surrounding the injection tract and three fields in each section were selected randomly for cell counting (n = 3 rats per group). *, **p < 0.05 and 0.01, respectively

To assess the levels of gene expression mediated by our lentiviral systems in the CNS *in vivo*, different combinations of vectors were injected into the hypoglossal motor nucleus of adult rats. All viral combinations were injected at a 4 : 1 ratio between the virus encoding the transactivator and the virus encoding the TRE-tight response element. Compared to the controls (Figures 4a and 4f), we observed approximately 12- and 8-fold increases in the number of DsRed2-positive cells in the rats injected with LV-Tretight-DsRed2/LV-2 × SYN-tTA and LV-Tretight-DsRed2/LV-2 × GfaABC_1_D-tTA, respectively (Figures 4b, 4g; 5A and 5B). The cells from groups injected with TA-containing vectors appeared much brighter, although this parameter was not quantified in this study. Please note that the density of DsRed2-positive astrocytes using LV-Tretight-DsRed2/LV-2 × GfaABC_1_D-tTA (Figure 5B) was higher than neurones using LV-Tretight-DsRed2/LV-2 × SYN-tTA (Figure 5A). This might reflect higher numbers of astrocytes compared to neurons *in vivo* but also be due to the higher transcriptional efficiency of the GfaABC_1_D promoter than that of the SYN promoter, because similar results were obtained *in vitro* (Figure 3).

To test the *in vivo* inducibility of gene expression, rats were injected with LV-Tretight-DsRed2/LV-2 × SYN-tTA or LV-Tretight-DsRed2/LV-2 × GfaABC_1_D-tTA and given drinking water in three different ways: (I) drinking water supplemented with 5% sucrose containing 2 mg/ml Dox for 7 days; (II) drinking water supplemented with 5% sucrose containing 2 mg/ml Dox for 7 days followed by normal drinking water for another 7 days; and (III) normal drinking water for 7 days followed by drinking water containing 2 mg/ml Dox in 5% sucrose for another 7 days. In group I, DsRed2 transgene expression was completely repressed by Dox (Figures 4c and 4h). In group II, DsRed2 expression became evident 4 days after the removal of Dox (data not shown) and reached the levels similar to those in the rats injected with the viruses but not treated with Dox by day 7 (Figures 4d and 4i). In group III, repression of DsRed2 expression became evident 4 days after the administration of Dox (data not shown) and only very few positive cells could be observed by day 7, most likely due to the very slow degradation of the pre-synthesised DsRed2 (Figures 4e and 4j). These results demonstrate efficient and reversible modulation of the transgene expression *in vivo*. Furthermore, consistent with our previous observations 22, we did not detect evidence of local inflammation, cell death or other pathology that could indicate toxicity of the TA-treated binary Tet systems.

### Cell-type specificity

**Figure 6:**
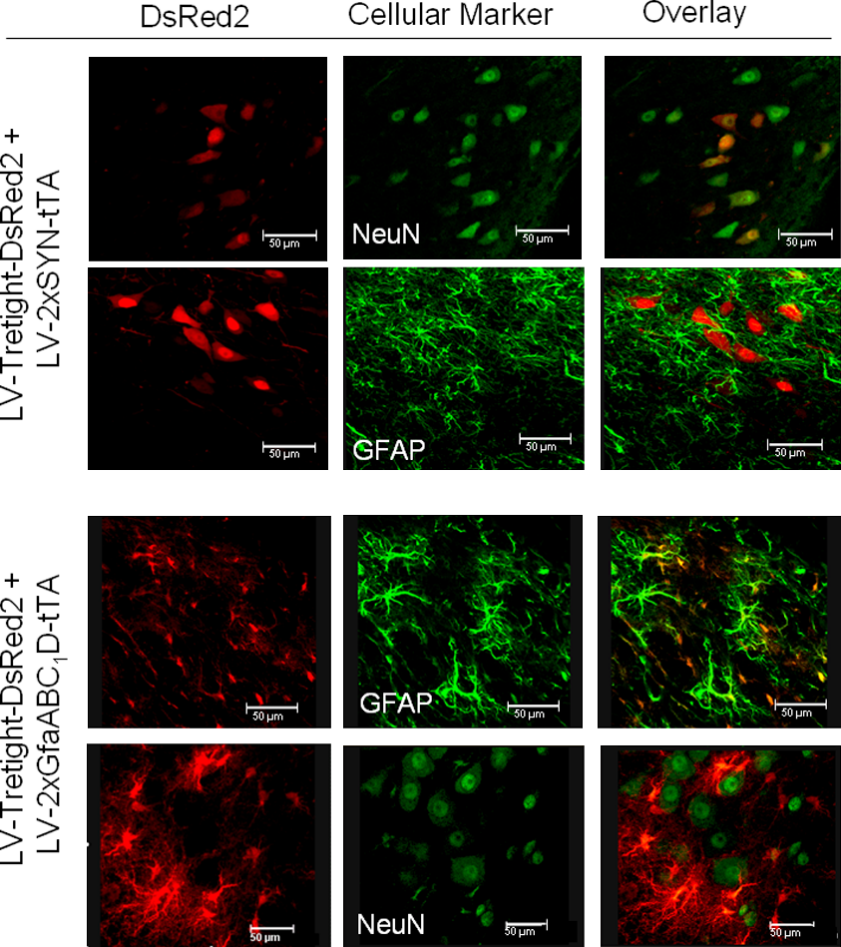
The specificity of TA-enhanced Tet-Off regulatory systems. Viruses were injected into the rat brain (see also legend for Figure 4). Seven days after injection, brain tissues were collected. Frozen coronal transverse 40 µm sections were cut and immunostained against NeuN to visualise neurons and against GFAP to visualise astroglial cells

One of the critical issues in applying the TA strategy using cell-type-specific promoters is whether their cell-type specificity is preserved. To address this issue, we performed immunohistochemistry using antibodies against neuron-specific nuclear protein (NeuN) to visualise neurons and antibodies against glial fibrillary acidic protein (GFAP) to visualise astrocytes (Figure 6). Essentially, all DsRed2-positive cells in the LV-Tretight-DsRed2/LV-2 × SYN-tTA-injected rats were NeuN-positive, whereas none stained positively for GFAP, indicating that the transgene was expressed exclusively in neurons. Conversely, in LV-Tretight-DsRed2/LV-2 × GfaABC_1_D-tTA-injected rats, the bulk of DsRed2-positive cells were positively stained with GFAP, while in no case was there co-localisation of DsRed2 and NeuN signals, demonstrating the restricted expression of DsRed2 to glial cells.

## Discussion

An expression system that can target specific cell types in the CNS and be tightly controlled by a drug would greatly facilitate neurobiological research and gene therapy applications. Tet-regulatable gene expression systems have the potential for regulatable targeted gene expression if coupled with cell-type-specific promoters. However, the application of tissue- or cell-type-specific promoters to target transgene expression is often hampered by their relatively weak activity. This disadvantage also compromised their use in the context of TRE-tight-based expression systems. We have previously demonstrated that TA is a potent strategy to augment transgene expression from two neuron-specific promoters in the brain *in vivo* without compromising their cell specificity 22. In view of the modular structure of the strategy, we reasoned that it should also work well for the enhancement of tTA expression using well-characterised but fairly weak mammalian promoters, such as SYN and GfaABC_1_D promoters. In the present study, we incorporated a TA step to potentiate tTA expression driven by these two cellular promoters. To our knowledge, this study is the first to incorporate the TA strategy in a tetracycline-regulatable cell-specific expression system. Transgene expression from this system is characterised by three beneficial features: cell-type-specific expression, a high level of expression and tight regulation by Dox.

The construction of the recombinant transcriptional activator, GAL4p65, is based on the well-identified modular structures of two transcriptional factors, the murine NFκB p65 and the yeast GAL4. NFκB is a group of dynamically modulated dimeric transcription factors, with the p50/p65 dimer being the most common protein complex regulating expression of mammalian genes across species, cell types and developmental stages. In the CNS, NFκB has a constitutive and relatively high level of activity in neurons 28,29 and plays a crucial role in neuronal survival in a variety of physiological and pathological settings 29–31. NFκB also plays a crucial role in regulating inflammatory gene expression in glial cells 32. The high efficiency of NFκB in neurons and glial cells indicates that co-activators and regulators required for NFκB transcriptional activity are present in these two groups of cells. The most active part of the transcription activation domain of NFκB p65 is located between amino acids 364 and 549 33, which have been used for generation of the mammalian two hybrid system by Stratagene. The yeast GAL4 gene expression system is one of the most widely studied eukaryotic transcriptional regulatory systems. Of the 881 amino acids that constitute the transcriptional activator of GAL4, the fragment compromising amino acids 1–147 contains the DNA-binding domain and also acts as a nuclear localisation signal 34. In this study, we fused the transcriptional activation domain of murine NFκB p65 (amino acids 364–550) to the DNA-binding domain of GAL4 (amino acids 1–147) to form GAL4p65. As demonstrated here, the generated chimeric protein works as a strong artificial transcriptional factor and provides significantly improved transgene expression.

In assessing the inducibility of our systems, an important consideration is that the dynamics of DsRed2 disappearance from the living cells will be strongly affected by the half-life of this protein while its ‘appearance’ will be affected by the speed of fluorophore maturation. Although we could not find the relevant information for DsRed2, its close analogue DsRed1 is believed to be very stable with a half-life of more than 4 days 35. *In vitro* experiments showed that transgene expression could be induced rapidly (4 days) following Dox withdrawal (Figures 2d and 2i). Furthermore, transgene expression could be repressed completely 4 days after the re-administration of Dox. The *in vitro* kinetics of gene expression of the Tet-Off system as described above are in agreement with previous studies 11,18. *In vivo* DsRed2 expression could also be switched on or off reversibly simply by adding or removing Dox from the drinking water. However, the time taken for transgene expression (∼7 days) to be turned on or off following the withdrawal or re-administration of Dox was significantly longer *in vivo* than *in vitro*. The relatively long *in vivo* half-life of Dox and its likely accumulation and release from tissues are the probable causes of these deferences 36,37.

To achieve well-preserved cell-type specificity, we chose to place the elements required for regulated expression, i.e, the TRE-tight element and the tTA, into two separate viral vectors. There is evidence suggesting that incorporating the transactivator and TRE promoter into the same vector decreases the efficiency of expression control and results in reduced ability to regulate gene expression and/or increased basal leakiness from the TRE promoter 19,21. We used ratios of 1 : 1 and 1 : 4 of virus of the TRE/DsRed2 reporter virus to the virus encoding the transactivator tTA. For *in vitro* experiments, a 1 : 1 ratio mediated effective transgene expression with no loss of regulatability. However, for *in vivo* experiments the ratio was changed to 1 : 4 as it gave better results than a 1 : 1 ratio (data not shown). Previous studies have reported varying results for the transactivator gene to the TRE/reporter gene ratio that produces optimal induction properties. While some authors found that a 1 : 1 tTA/TRE ratio gave optimal induction and negligible background expression, others found that higher or lower ratios were optimal 19,21,38–40. Multiple variables could contribute to these different findings, including the promoters used for the expression of the transactivator, the cell lines, or *in vivo* target organ, the delivery system used and the sensitivity of the detection method for levels of transgene expression. It is therefore important to establish the optimal conditions for each application or target organ.

Placing tTA and TRE-tight into separate vectors also has the additional advantage of extending the use of a given construct by matching it with different partners. One particularly exciting application of this strategy is cell-specific targeting of TRE-tight-controlled miRNA-expressing cassettes for gene knock-down, as described by Stegmeier *et al.* 41. On the other hand, a potential drawback of the binary system is that only the cells dually transduced with both viral vectors will express the desired transgene. The apparent inability to efficiently co-transduce different virions into the same cell efficiently has been attributed to viral interference 42, which can occur between two dissimilar or homologous viruses 43,44. The mechanism underlying the interference is unclear, although it might occur at the level of viral entry through receptor down-regulation or the ability of one virus to inhibit the replication of a second virus within the same cell 45–47. However, successful co-transduction of both dividing and non-dividing cells with two VSV-G-pseudotyped HIV-1 vectors is possible 48. The efficiency of the co-transduction was directly proportional to the individual transduction efficiencies obtained with single vectors. It was hypothesised that VSV-G pseudotyping would preclude receptor interference based on the observation that VSV-G-mediated entry is receptor-independent 45,49. We did not specifically quantify the co-transduction efficiency in this study. Since TRE-tight-driven DsRed2 expression was essentially undetectable in the absence of a second tTA-expressing vector, it is clear that our binary VSV-G-pseudotyped LV vectors successfully co-tranduced both neuronal and astroglial cells *in vitro* and *in vivo*.

In conclusion, we present a new strategy to overcome the problem of insufficient activity of cell-specific promoters in the context of TRE-tight gene expression vectors. This represents a significant step towards developing effective gene delivery systems that can be used for diverse applications, ranging from basic biomedical investigations to cell-type-specific gene therapy of numerous brain disorders.
